# Universal Single-Probe RT-PCR Assay for Diagnosis of Dengue Virus Infections

**DOI:** 10.1371/journal.pntd.0003416

**Published:** 2014-12-18

**Authors:** Erik Alm, Birgitta Lesko, Gunnel Lindegren, Clas Ahlm, Sandra Söderholm, Kerstin I. Falk, Nina Lagerqvist

**Affiliations:** 1 Department of Microbiology, Public Health Agency of Sweden, Solna, Sweden; 2 Department of Laboratory Medicine, Karolinska Institutet, Stockholm, Sweden; 3 Department of Clinical Microbiology, Umeå University, Umeå, Sweden; 4 Department of Microbiology, Tumor and Cell Biology, Karolinska Institutet, Stockholm, Sweden; 5 Department of Medicine, Karolinska Institutet, Karolinska University Hospital Huddinge, Stockholm, Sweden; The Connecticut Agricultural Experiment Station, United States of America

## Abstract

**Background:**

Dengue is a mosquito-borne viral disease that has become more prevalent in the last few decades. Most patients are viremic when they present with symptoms, and early diagnosis of dengue is important in preventing severe clinical complications associated with this disease and also represents a key factor in differential diagnosis. Here, we designed and validated a hydrolysis-probe-based one-step real-time RT-PCR assay that targets the genomes of dengue virus serotypes 1–4.

**Methodology/Principal Findings:**

The primers and probe used in our RT-PCR assay were designed to target the 3′ untranslated region of all complete genome sequences of dengue virus available in GenBank (n = 3,305). Performance of the assay was evaluated using in vitro transcribed RNA, laboratory-adapted virus strains, external control panels, and clinical specimens. The linear dynamic range was found to be 10^4^–10^11^ GCE/mL, and the detection limit was between 6.0×10^2^ and 1.1×10^3^ GCE/mL depending on target sequence. The assay did not cross-react with human RNA, nor did it produce false-positive results for other human pathogenic flaviviruses or clinically important etiological agents of febrile illnesses. We used clinical serum samples obtained from returning travelers with dengue-compatible symptomatology (n = 163) to evaluate the diagnostic relevance of our assay, and laboratory diagnosis performed by the RT-PCR assay had 100% positive agreement with diagnosis performed by NS1 antigen detection. In a retrospective evaluation including 60 archived serum samples collected from confirmed dengue cases 1–9 days after disease onset, the RT-PCR assay detected viral RNA up to 9 days after appearance of symptoms.

**Conclusions/Significance:**

The validation of the RT-PCR assay presented here indicates that this technique can be a reliable diagnostic tool, and *hence* we suggest that it be introduced as the method of choice during the first 5 days of dengue symptoms.

## Author Summary

Dengue is the most common mosquito-borne viral disease affecting humans, with an estimated 100 million cases annually. Being able to achieve early and correct detection of all four serotypes of dengue virus can have an impact on the diagnosis of individual patients, such as febrile travelers returning home, but can also be useful in endemic countries, particularly during the early stage of an outbreak. After analyzing all the complete genome sequences of dengue virus serotypes 1 to 4 published in GenBank, we constructed a one-step real-time RT-PCR assay that can be used in different settings to detect dengue virus in clinical samples. Extensive evaluation of the performance of this assay by testing samples from dengue patients confirmed the usefulness of the method in detecting early stage infection. In short, our dengue virus RT-PCR proved to be a reliable diagnostic tool and a relevant alternative and/or complement to NS1 antigen-detecting tests and serological methods for diagnosis of acute dengue.

## Introduction

Dengue has gradually become one of the leading causes of morbidity in tropical and subtropical regions [1], and this disease is caused by infection with any of four genetically related dengue virus (DENV) serotypes (designated 1 to 4). DENV has a positive-stranded RNA genome and belongs to the family *Flaviviridae* and the genus *Flavivirus*. The geographical areas in which DENV circulates have expanded in recent years, and all four serotypes are now found in Asia, Africa, and the Americas [2]. Endemic dengue has been reported from more than 100 countries [1], many of which are popular tourist destinations. Thus diagnostic challenges include both patients residing in endemic countries and travelers returning home with fever after visiting such regions.

Most DENV infections are asymptomatic or are manifested by a sudden onset of fever 4 to 6 days after infection [3] that is accompanied by nonspecific signs and symptoms such as nausea, headache, rash, and muscle and joint pain, without any severe sequelae [4]. However, some patients develop dengue with severe symptoms, which was formerly termed dengue hemorrhagic fever or dengue shock syndrome [5]. This critical phase begins at defervescence and is characterized by plasma leakage leading to respiratory distress and shock, internal hemorrhage, and organ impairment, manifestations that can have a deadly outcome if proper clinical management is delayed [6].

Infection with one serotype confers long-term protective immunity against that particular serotype only, whereas subsequent infection with a heterologous serotype may predispose a person to develop severe disease [7]. It is not fully understood why some individuals resolve DENV infections without any complications, whereas others develop severe symptoms, and no single factor has yet been identified as the sole contributor to disease severity [8]. It has been suggested that the infecting DENV subtype or strain influences disease outcome [9], [10], but the genetic characteristics of the infecting virus are currently not taken into account in the management and clinical care of acutely ill patients [6].

Considering the broad spectrum of clinical presentations of dengue, as well as the differential diagnosis to other diseases, laboratory confirmation is essential. Fever is the most common presenting symptom and often coincides with viremia [11] and non-structural protein 1 (NS1) antigenemia [12], [13]. NS1 is a soluble protein, and it has been shown to be present in serum for up to nine days after onset of fever in a proportion of DENV-1 infected patients [14] and is therefore the target for several diagnostic tests. Ever since these assays were introduced, they have been widely used for early dengue diagnosis [15]–[17]. Unfortunately, recent evaluations have shown that although the NS1 antigen detection tests offer high specificity [18], [19], they differ markedly in sensitivity [18], [20]–[22].

Serological diagnosis is usually achieved by detecting DENV-specific IgM and IgG antibodies, which start to appear during the early post-febrile period [23]. A primary infection is characterized by slow and low-titer antibody responses, and IgM antibodies represent the dominant isotype. IgM antibodies appear by day 2 to 5 of illness [24], [25], whereas IgG antibodies generally become detectable at the end of the first week after onset of symptoms [2]. The opposite is typically observed in secondary infections: IgM antibody levels are undetectable or low, and IgG antibody titers rise rapidly [24]. Consequently, laboratory diagnosis based entirely on detection of IgM antibodies can give false-negative results in patients that have previously been infected with DENV [24]. Unspecific reactions are also an important issue to consider in dengue serodiagnosis. Commercially available tests for rapid detection of IgM antibodies have been shown to produce false-positive results in sera positive for anti-DENV IgG, malaria, and rheumatoid factor [26], and assays detecting anti-DENV IgG antibodies can be vulnerable to cross-reactions caused by antibodies against related viruses [3].

Due to the mentioned limitations of the conventional assays, the use of molecular methods to detect viral genomes for early diagnosis of dengue has increased. Several specific and sensitive PCR-based assays have been developed during the past years [27]–[34], and one DENV serotype-specific multiplex real-time RT-PCR has been approved by the US Food and Drug Administration for use in clinical settings [35]. Some of those assays are less sensitive gel-based PCR methods [32], [33], whereas there are other techniques such as the multiplex real-time RT-PCR that require advanced thermal cyclers that detect the four specific fluorophores for which these systems have been validated [28], [31], [35]. The common denominator for most of these assays is the large number of primers and/or probes that are needed to detect the genomes of the four DENV serotypes [27], [28], [30], [31], which may complicate modifications of primer and probe sequences when new genetic variants of DENV emerge. Accordingly, we developed a new universal DENV RT-PCR assay that has a single fluorophore-labeled hydrolysis probe and three primers, and represents a system designed to target the 3′ untranslated region (UTR) of all complete genome sequences (n = 3,305) of the four DENV serotypes that have been published thus far. We used in vitro transcribed RNA, laboratory-adapted virus strains, external control panels, and clinical samples to evaluate the DENV RT-PCR assay, and the results showed that the method is highly sensitive and specific for early diagnosis of suspected dengue.

## Methods

### Ethics statement and clinical specimens

Serum samples were obtained from the biobank repository of the Public Health Agency of Sweden (formerly the Swedish Institute for Communicable Disease Control) as stipulated in the regulations for use of such material in diagnostic development and quality assessment. The Swedish Ethical Review Act (2003:16), Ethical Review of Research Involving Humans (http://www.epn.se/media/45159/the_etical_review_act.pdf) is not applicable, for this reason, no informed consent was required. These samples were used for diagnostic development and quality assessment.

During January and February 2014, sera (n = 163) were collected from all patients who had returned from travel abroad showing dengue-compatible symptomatology and epidemiology. Information on day of disease onset and travel history was available in most cases. Furthermore, serum samples that had been obtained from confirmed dengue patients (n = 60) during the years 2011–2013 were analyzed retrospectively; the day of disease onset was known for these patients. Clinical specimens from patients with non-dengue diagnoses were collected during 2014.

### Viruses and external control panels

The specificity of the DENV RT-PCR assay was evaluated by testing RNA extracted from preparations of the following: DENV-1 (strains West Pac., Hawaii, and 8356/10), DENV-2 (strains NewGuinea C. and 4397/11), DENV-3 (strains H-87 and 3140/09), DENV-4 (strains H-241 and 3274/09), other human pathogenic members of the *Flaviviridae* family (Japanese encephalitis virus [JEV; strain Nakayama], tick-borne encephalitis virus [TBEV; strains Hochosterwitz, Sofjin, and Latvia], West Nile virus [WNV; strains Eg101, MgAn 786/6/1995, WN_0304, and Ug_1937], Zika virus [ZIKV; strain MR766], Usutu virus [USUV; strain g39], and yellow fever virus [YFV; strain Asibi]), and six non-flaviviruses representing the three virus families *Togaviridae* (chikungunya virus [CHIKV; strains 23161 and Malaysia], *Areanaviridae* (Lassa virus [LASV; strain Josiah]), and *Bunyaviridae* (Rift Valley fever virus [RVFV; strain ZH548], Dobrava virus [DOBV; strain H119/99], Hantaan virus [HTNV; strain 76–118], and Seoul virus [SEOV; strain R22]). Conditions for virus propagation are described in S1 Text. Handling of infectious material was performed in biosafety level 3 or 4 containment laboratories depending on classification of each virus.

Three external DENV control panels were obtained from Quality Control for Molecular Diagnosis (QCMD, http://www.qcmd.org/) in the years 2011, 2012, and 2013. These panels consisted of 12 samples each: 10 containing DENV serotypes 1–4 and two control samples.

### RNA extraction

Total RNA was extracted from 140-µL aliquots of clinical specimens or supernatant of infected cells using a QIAamp viral RNA mini kit (Qiagen) according to the protocol suggested by the manufacturer. The RNA was eluted with 60 µL of elution buffer and stored at −80°C pending analysis. RNA extracted from supernatants of virus-infected cells was assayed at a dilution of 1∶10 or 1∶20.

### Design of primers and probe

To find the most conserved region in the DENV genome, all whole genome sequences that were available at the design stage (November 1, 2013) were downloaded from the National Center for Biotechnology Information (NCBI) and used for assay design. Multiple sequence alignments containing all genomic sequences were created using Clustal Omega v 1.2.0 [36]. Primers and probes were designed using in-house software capable of optimizing sequence conservation and estimating chemical properties of the oligonucleotides simultaneously. Melting temperatures (Tm) were verified using Primer Express v3.0 (Applied Biosystems, LifeTechnologies). The theoretical specificity of the system was investigated using BLAST against the NCBI nucleotide database.

### One-step real-time RT-PCR

The DENV one-step real-time RT-PCR assay was carried out in 25 µL of reaction mixture containing 5 µL of RNA template, TaqMan Fast Virus 1-Step Master Mix (Life Technologies), nuclease-free H_2_O (Quanta), each primer at 0.9 µM, and 0.2 µM probe. The primers and probe were purchased from Life Technologies, and their sequences are presented in Table 1. The minor-groove-binding (MGB)-probe was labeled with 6-carboxy fluorescein (FAM) reporter dye and a non-fluorescent quencher (NFQ). Amplification and detection of the 64-nucleotide amplicon were performed in a StepOne Plus real-time PCR system (Applied Biosystem, Life Technologies). Thermal cycling parameters were as follows: reverse transcription at 50°C for 5 min, inactivation at 95°C for 20 s followed by 40 cycles of fluorescence detection at 95°C for 3 s, and annealing at 60°C for 30 s. The baseline and threshold were set using the auto-baseline and threshold feature in StepOne Software v2.2.2 (Life Technologies). Samples were considered negative (not determined) if no target amplification was recorded after 40 cycles. Samples were tested in duplicate if not stated otherwise. The concentration of detected virus RNA or in vitro transcribed RNA was measured in genome copy equivalents (GCE)/mL. To ensure adequate RNA extraction from clinical samples, mRNA of human beta-actin was assessed using a TaqMan probe-based RT-PCR assay (Life Technologies). Dilutions and pipetting were performed using a robotic QIAgility workstation (Qiagen). The CDC DENV-1-4 Real-Time RT-PCR Assay was performed in singleplex reactions according to the manufacturer's instructions (Centers for Disease Control and Prevention). Amplification and detection were performed in a 7500 Fast DX Real-time PCR instrument (Applied Biosystems).

**Table 1:** Characteristics of the primers and probe targeting the 3′ UTR of DENV serotypes 1–4.

Name	Sequence (5′-3′)	Position^1^	Tm^2^
DENV_F	GCATATTGACGCTGGGARAGAC	10632–10653	59.7
DENV_R1-3	TTCTGTGCCTGGAATGATGCTG	10674–10695	63.1
DENV_R4	YTCTGTGCCTGGATWGATGTTG	10674–10695	63.1
DENV_P	6FAM-CAGAGATCCTGCTGTC-MGB(NFQ)	10654–10669	67.0

1 Genome positions are given according to the DENV-1 NCBI reference sequence (GenBank acc. no. NC_001477).

2 The mean melting temperature (Tm) is shown for degenerate primers (R, A/G; W, A/T; Y, G/A/C).

6FAM = 6-carboxy fluorescein; MGB = minor-groove-binding; NFQ = non-fluorescent quencher.

### Evaluation of RT-PCR assay performance

The dynamic range and the efficiency of amplification of the DENV RT-PCR assay were determined by assaying triplicates of 10-fold serial dilutions of in vitro transcribed RNA (BioSynthesis Inc.). The RNA transcripts (denoted RNA[DENV_1–3] and RNA[DENV_4]) were 88 and 84 nt, and were based on the sequences of DENV-1 (GenBank acc. no. FJ687428) and DENV-4 (GenBank acc. no. FJ882597) complementary to the DENV_R1–3 and DENV_R4 primers, respectively. The sequences of the in vitro transcribed RNA can be found in S2 Text.

The limit of detection (LoD) was determined by assaying twofold serial dilutions close to the detection limit of the assay of in vitro transcribed RNA. The RNA transcripts were tested in eight replicates in three separate experiments. LoD was defined as the last dilution in which RNA was detected in all replicates.

### Gel-based RT-PCR and sequencing

DENV-specific RNA was amplified in 25-µL reaction mixtures by primer pairs 5′-TCAATATGCTGAAACGCGHGAG-3′ and 5′-GCGCCTTCNGNNGACATCCA-3′ using a OneStep RT-PCR kit (Qiagen). These primers target positions 132–153 and 764–783 of the DENV-1 NCBI reference sequence (GenBank acc. no. NC_001477). The reaction mixtures were placed in a Veriti Thermal Cycler (Applied Biosystems) and were cycled at 50°C for 30 min, 95°C for 15 min, followed by 50 cycles of 95°C for 20 s, 50°C for 30 s, and 70°C for 1 min, and a final extension for 10 min at 72°C. The amplified product (652 nt) was analyzed by electrophoresis and purified using a QIAquick PCR Purification kit (Qiagen). The sequence was determined by bi-directional Sanger sequencing (GATC Biotech) and compared with sequences in GenBank using BLASTn.

### Antigen and antibody detection methods

Soluble NS1 DENV antigen was detected using the immunochromatographic one-step assay SD Bioline Dengue NS1 Ag Rapid Test (Standard Diagnostics Inc.) according to the manufacturer's protocol. The presence of DENV-specific IgM and IgG antibodies in clinical serum samples was determined by Panbio Dengue IgM capture ELISA (Alere) and an in-house immunofluorescence assay (IFA) [37], respectively. The IgM assay was performed according to the manufacturer's guidelines, and a result was considered positive if>11 pan-bio units (PBU) were obtained. IFA was performed according to a previously published protocol [37], and a titer of ≥40 was regarded as a positive result. The appropriate assay/assays were selected based on the time elapsed after onset of symptoms as reported by the clinician. Inasmuch as no single assay can correctly diagnose all dengue patients at the various times they may seek medical attention, a diagnosis of “confirmed dengue” was reached based on positive results in at least two of three assays (ELISA, IFA, or NS1 antigen detection), or a fourfold rise in IgG titers between paired sera (collected at least 7 days apart).

## Results/Discussion

### Assay design

The DENV one-step real-time RT-PCR assay was designed to target all complete genome sequences of DENV serotypes 1–4 available in GenBank at the time (November 1, 2013). The dataset used to design the primers and probe comprised a total of 3,305 DENV genomes representing 1,735 DENV-1, 929 DENV-2, 517 DENV-3, and 124 DENV-4 sequences. These features distinguish our assay from other published PCR assays, most of which are developed based on a selected number of sequences or designed to detect currently circulating DENV strains [27], [28], [30], [34], [35], [38]. However, a study showing simultaneous circulation of new and old strains has highlighted the importance of being able to detect diverse strains of this virus [39]. Furthermore, we included 12 sylvatic DENV sequences in our dataset. This was done because there have been observations of spillover of sylvatic DENV to humans [40]–[42], which has resulted in small outbreaks or individual cases of severe dengue.

Very few regions in the DENV genome are sufficiently conserved for a universal DENV RT-PCR system, and the variability of the entire genome is shown in S1 Figure in the Supporting Information. A candidate region with relatively high sequence conservation was identified in the 3′ UTR genomic positions 10632–10695 (numbering relative to DENV-1, GenBank acc. no. NC_001477) (S1 Figure). This region is large enough to accommodate a TaqMan system with a short MGB-modified probe.

**Figure 1:**
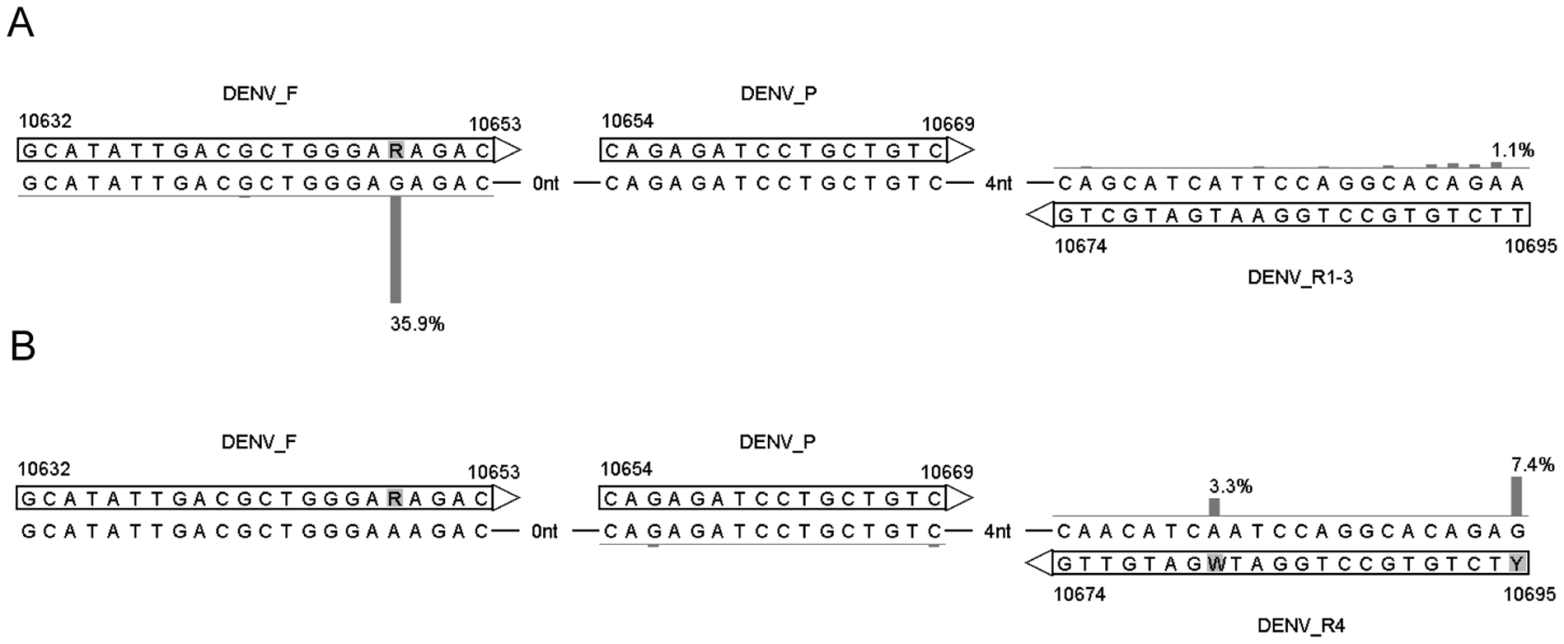
Overview of the primers and probe in the DENV RT-PCR assay. The reverse primers DENV_R1–3 (A) and DENV_R4 (B) were specifically designed to target DENV serotypes 1–3 and serotype 4, respectively. Vertical bars and percentages show the fraction of sequences with nucleotides deviating from the consensus of DENV serotypes 1–3 (A) and serotype 4 (B). Percentages below 1 are not shown. Numbers indicate genomic positions.

The region 10654–10669 is exceptionally conserved; three of the 3,305 genomes studied were found to contain two mutations in this region (GenBank acc. no. JQ922554, FJ639735, and FJ639819), and only nine genomes contained a single mutation. One of the sequences with two mutations was collected in 1963 (JQ922554) and the indels in the 3′ UTR were not confirmed according to the authors for the remaining two sequences (FJ639735 and FJ639819). For this reason, and because the surrounding sequence could accommodate both forward and reverse primers in conserved regions when using only a few ambiguous nucleotides, we designed the DENV_P TaqMan probe to target the 10654–10669 region. DENV-4 differs from the other serotypes at positions 10670–10695. Consequently, the DENV_R4 primer was designed to specifically target DENV-4, whereas the DENV_R1–3 primer was designed to target DENV-1, -2, and -3. The primers and probe sequences and positions are shown in Fig. 1, in which deviations from the consensus sequence are indicated by vertical bars and percentages of the total dataset. S2 Figure in the Supporting Information shows the geographic and temporal distributions of the sequences of the four DENV serotypes and their deviations from the primer and probe sequences. The 3′ UTR of DENV has a complex secondary structure [43]. The secondary structure at the primer and probe binding site is shown in S3 Figure in the Supporting Information.

The assay target site (length 64 nt) was matched against all non-DENV sequences in the NCBI nucleotide database by using BLASTn with very loose match criteria (word-size = 7, E-cutoff = 1,000, match/mismatch cost +1/−1, Gap cost 5/2). The hits with the lowest E-value were ZIKV. These ZIKV sequences (GenBank acc. no. KF268950, KF268949, and KF268948) matched only the forward primer and a part of the probe-binding site, hence it is very unlikely that any non-specific amplification will occur when using this assay. An alignment showing the 3′ UTR sequences of pathogenic flaviviruses is included in the Supporting Information, S1 File.

### Assay performance

The performance of the DENV RT-PCR assay was evaluated using a known concentration of in vitro transcribed RNA. In this assessment, we used two RNA transcripts, RNA[DENV_1–3] and RNA[DENV_4], which accommodate binding for reverse primer DENV_R1–3 and DENV_R4, respectively.

**Figure 2:**
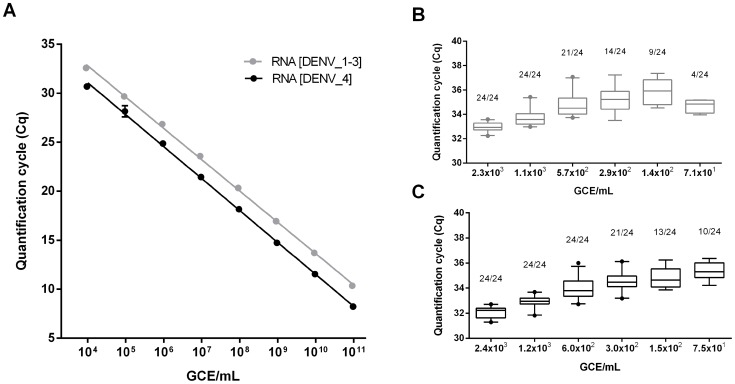
Dynamic range and limit of detection of the DENV RT-PCR assay. (A) The linear dynamic range of the DENV RT-PCR assay was determined by testing triplicates of 10-fold serially diluted in vitro transcribed RNA. The sequences of the transcript RNA (RNA[DENV_R1–3] and RNA[DENV_R4]) were matched with the two reverse primers DENV_R1–3 and DENV_R4, respectively. Each dot represents the mean Cq-value from three replicates, the error bars indicate the 95% confidence interval, and the lines illustrate the result of the lin-log regression analysis. (B and C) Limit of detection was determined by assaying eight replicates of twofold serially diluted RNA transcripts in three separate experiments, and the results of testing are shown for RNA[DENV_R1–3] (B) and RNA[DENV_R4] (C). Horizontal lines indicate mean values, boxes denote the 25th to 75th percentiles and whiskers the 5–95% percentiles, and dots represent outliers. The number of positives per total number of replicates tested is given above each box. Limit of detection was defined as the last dilution in which transcript RNA was detected in all 24 replicates. GCE = genome copy equivalents.

The linear dynamic range and the efficiency of amplification were determined by assaying triplicates of 10-fold dilutions of transcript RNA (Fig. 2A). The DENV RT-PCR assay could distinguish differences in concentration over a range of 9.6×10^3^ to 9.6×10^10^ GCE/mL RNA[DENV_1–3] and, similarly, 9.1×10^3^ to 9.1×10^10^ GCE/mL RNA[DENV_4] (Fig. 2A). Values representing the efficiency of amplification for the corresponding template concentrations were 105.6% (R^2^ = 0.999, y-intercept 39.4) and 102.6% (R^2^ = 0.999, y-intercept 37.8), respectively (Fig. 2A). The intra- and inter-assay coefficients of variation at 4.6×10^3^ GCE/mL RNA[DENV_1–3] were 0.8% and 1.0%, respectively, and corresponding coefficients at 4.8×10^3^ GCE/mL RNA[DENV_4] were 0.5% and 1.4%.

LoD was determined empirically by testing twofold serial dilutions of transcript RNA in the analytical range of the expected detection limit. The DENV RT-PCR assay could detect all 24 replicates of 1.1×10^3^ GCE/mL (corresponding to approximately 14 GCE/reaction) RNA[DENV_1–3] and 24 replicates of 6.0×10^2^ GCE/mL (approximately 7 GCE/reaction) RNA[DENV_4] (Fig. 2, B and C).

To determine the performance of the DENV RT-PCR assay in common matrices associated with dengue laboratory diagnostics [6], [44], we compared the slopes of the standard curves generated by amplification of RNA extracted from DENV spiked in human serum, plasma, and cerebrospinal fluid (CSF) with the slopes of such curves for RNA extracted from DENV diluted in water. Due to the use of serotype-specific reverse primers, we included DENV-1 and DENV-4 in the analysis. Depending on the type of co-extracted inhibitor, a variety of inhibition mechanisms can occur during the PCR process [45]. By diluting the RNA, and thereby diluting the co-extracted inhibitor, the negative effect of inhibition in different matrices can be detected. With the exception of DENV-4 diluted in plasma (p = 0.036), the slopes of the lin-log standard curves generated from RNA diluted fivefold were not significantly different from the slopes obtained from viral RNA extracted from water (DENV-1 in serum p = 0.62, plasma p = 0.05, and CSF p = 0.38; DENV-4 in serum p = 0.77 and CSF p = 0.69; two-way ANOVA, three replicates), which indicates that, in general, PCR inhibition was minimal in samples of serum, plasma, and CSF.

### Assay specificity

Three external control panels obtained from QCMD were used to evaluate the DENV RT-PCR assay regarding its capacity to detect all four DENV serotypes (S1 Table). Each panel consisted of ten samples containing various concentrations of DENV (10^3^ to 10^6^ GCE/mL), a non-DENV flavivirus control, and one negative control. Independent of genome copy number, the DENV RT-PCR assay detected all samples containing DENV, and no unspecific reactions were observed in control samples (Table S1).

To exclude possible cross-reaction between the DENV RT-PCR assay and human RNA, we analyzed 46 serum samples collected from patients with non-dengue diagnoses. None of these samples tested positive in the DENV RT-PCR assay (Table 2), whereas all were found to be positive for beta-actin mRNA, thus indicating adequate RNA extraction. To analyze the ability of the DENV RT-PCR assay to detect DENV in clinical samples, we tested 15 serum samples obtained from patients who had presented with dengue-compatible symptomatology and were confirmed to be DENV positive by sequencing. Of these samples, eight were positive for DENV-1, three for DENV-2, and four for DENV-3, and the DENV RT-PCR assay detected the DENV in all of those samples (Table 2). Unfortunately, we were unable to locate serum samples known to be positive for DENV-4.

**Table 2:** DENV RT-PCR assay specificity.

Sample	No. positive (no. tested)
Clinical samples (not dengue)	0 (46)
DENV-1 clinical samples^1^	8 (8)
DENV-2 clinical samples^1^	3 (3)
DENV-3 clinical samples^1^	4 (4)
DENV-1^2^	3 (3)
DENV-2^2^	2 (2)
DENV-3^2^	2 (2)
DENV-4^2^	2 (2)
Non-DENV flaviviruses^2^	0 (11)
Non-flaviviruses^2^	0 (7)

1 The DENV serotype was determined by Sanger sequencing.

2 Laboratory-adapted strains of flavivirus (DENV, JEV, TBEV, USUV, WNV, YFV, and ZIKV) and non-flavivirus (CHIKV, DOBV, HTNV, LASV, RVFV, and SEOV) are specified in Methods.

Potential cross-reactivity with genetically related viruses was evaluated by assaying the nucleic acid of six human pathogenic flaviviruses, which were represented by a total of 11 strains (Table 2). In view of the non-specific clinical presentation and course of dengue, differential diagnosis of this disease should also take several other conditions into consideration [46]. These include infections with CHIKV and RVFV, which are etiological agents of febrile illnesses that have been reported to have habitats that partially overlap those of DENV [47]. Accordingly, we also assessed our DENV RT-PCR method by assaying RNA of CHIKV and RVFV and other viruses. The primers and probe did not detect the genomes of non-DENV flaviviruses or non-flaviviruses (Table 2).

### Clinical performance

**Figure 3:**
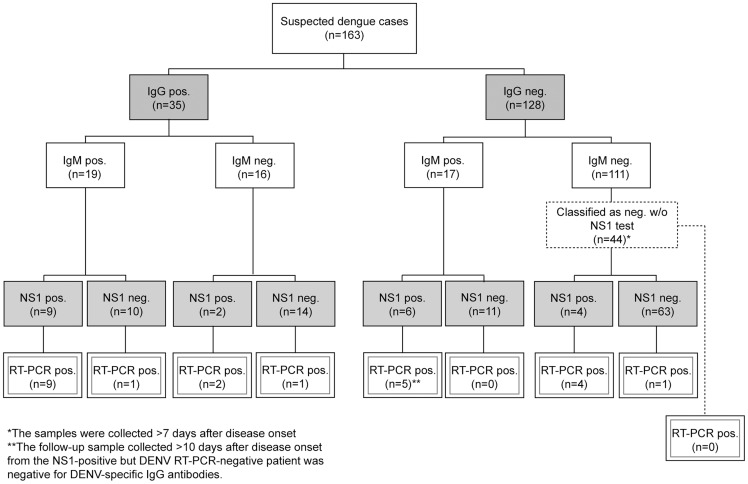
Flow-chart showing the laboratory test results for samples from returning travelers with dengue-compatible symptomatology. Serum samples collected during January and February 2014 were tested consecutively by the newly developed DENV RT-PCR method, an NS1 antigen detection test, IgM capture ELISA, and/or an in-house IFA detecting DENV-specific IgG antibodies. The criteria for a positive result in each individual assay are explained in Methods. The results of the laboratory analysis of the first sample arriving at the Public Health Agency of Sweden are shown. w/o = without.

We evaluated the clinical performance of the DENV RT-PCR assay by analyzing serum samples from residents of Sweden who presented with febrile illness after returning from travel to dengue-endemic regions. The serum samples were obtained from January to February 2014 and were tested consecutively using our DENV RT-PCR assay, the NS1 antigen rapid test, IgM capture ELISA, and/or in-house IFA-detecting anti-DENV IgG antibodies (Fig. 3). The integrity of the extraction reagents and the successful recovery of RNA from the analyzed patient serum samples were verified by detection of beta-actin mRNA.

We used the novel DENV RT-PCR method to analyze a total of 163 serum samples from patients with suspected dengue, and the assay detected viral RNA in 23 of the samples (Fig. 3). Ten of the samples that were positive by DENV RT-PCR contained detectable levels of both IgM and IgG antibodies, indicating that this assay is still useful as a diagnostic tool in patients presenting with anti-DENV-specific antibodies. Only 20 of the 23 samples that were positive by the DENV RT-PCR assay were also positive by the NS1 antigen test, indicating low sensitivity of the latter method. The three samples that were only positive in the DENV RT-PCR were also confirmed positive using the CDC DENV-1-4 RT-PCR Assay. One sample that tested positive solely for NS1 antigen (faint line) was not positive for DENV in our assay, and the convalescence sample from this patient (collected more than 10 days after disease onset) did not contain detectable levels of anti-DENV IgG antibodies (Fig. 3). These results strongly suggest a false-positive result in the NS1 antigen test.

Three samples tested negative for IgM antibodies but were positive for viral RNA and IgG antibodies (titers 40, 40, and 2,860; Fig. 3), which implies secondary infections or false-negative results in the IgM capture ELISA. Eleven serum samples were found to be positive for DENV IgM antibodies, although no other method could confirm an acute dengue infection. The low specificity of commercial IgM tests has been highlighted in a previous study in which false-positive results were observed in samples from both patients with other infections and healthy donors [48].

To summarize, in six acutely ill patients who would not have been confirmed as having dengue based on analysis of their first serum sample by use of the diagnostic algorithm applied in our laboratory (i.e., positive in two out of three tests), the virus was detected by our DENV RT-PCR assay. One of these DENV RT-PCR-positive patients also had detectable IgG antibody levels, four were also positive for NS1 antigen but not IgG or IgM, and the sixth patient was negative in all other tests (Fig. 3). These six samples were confirmed DENV positive using the CDC DENV-1-4 Real Time RT-PCR Assay, whereof two contained DENV-2, two contained DENV-3, and two samples contained DENV-4.

### Diagnostic application

**Figure 4:**
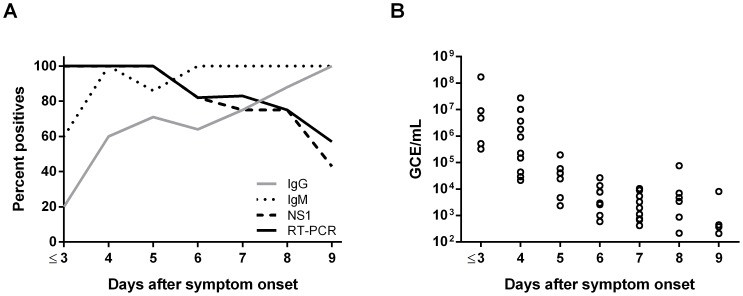
Results of the DENV RT-PCR assay performed on serum samples obtained 1 to 9 days after onset of symptoms. (A) Archived serum samples that had been collected from 60 patients on days 1–3 (n = 5), 4 (n = 10), 5 (n = 7), 6 (n = 11), 7 (n = 12), 8 (n = 8), and 9 (n = 7) after disease onset were tested by the DENV RT-PCR assay, an NS1 antigen detection test, IgM capture ELISA, and IFA detecting DENV-specific IgG antibodies. The criteria for a positive result in each of these analyses are explained in Methods. The curves show the percent of samples testing positive in the individual assays for the specified days after disease onset. (B) Viral load in samples collected on days 1–9 after onset of symptoms. Each dot represents the mean of results for duplicate samples from a single patient. GCE = genome copy equivalents.

The DENV RT-PCR assay was further evaluated using 60 archived serum samples that were collected in 2012 and 2013 from returning travelers for whom a dengue diagnosis was confirmed and the day of disease onset was reported (Fig. 4). The samples represented the following amounts of time elapsed after onset of symptoms: 1–3 days (n = 5), 4 days (n = 10), 5 days (n = 7), 6 days (n = 11), 7 days (n = 12), 8 days (n = 8), and 9 days (n = 7). The laboratory diagnosis was performed as described in Methods (Fig. 4A). For the patients who had provided samples 1–3 days after disease onset, analysis of a second covalence sample confirmed the dengue diagnosis. To determine the approximate virus levels in samples collected 1–9 days after symptoms appeared, we included a standard containing 10^3^ to 10^8^ GCE/mL RNA[DENV_R1–3] (Fig. 4B). When possible, the infecting serotypes of the DENV positive samples were determined using the CDC DENV-1-4 Real-Time RT-PCR Assay, for detailed information see S2 Table in the Supporting Information.

There was 100% positive agreement [49] between the DENV RT-PCR assay and NS1 antigen detection, and the DENV RT-PCR method revealed viral RNA in two additional samples in which no NS1 antigen could be detected (Fig. 4). Furthermore, the DENV RT-PCR assay detected viral RNA in all samples up to 5 days after disease onset (Fig. 4A), and a detection rate of at least 70% has previously been reported for RT-PCR in this time interval [12], [28], [50]. It is generally accepted that when DENV IgM levels become detectable, the viral load will have declined below measureable levels [6]. Nevertheless, in our study, 82% and 57% of the samples collected 6 and 9 days, respectively, after disease onset tested positive for viral RNA by the DENV RT-PCR assay, even though all patients had seroconverted (IgM and/or IgG) at the indicated time points (Fig. 4A). Similar to the results presented in 2008 by Dumoulin et al. [51], we were unable to detect DENV in samples obtained 10 days after appearance of symptoms.

### Concluding remarks

Here we present an alternative to the more complicated multiplex RT-PCR assays for detection of DENV serotypes 1–4. Having the capacity to detect a wide range of genetic DENV variants would enable diagnostic laboratories to resolve traveler-associated infections in non-endemic countries and to quickly respond to outbreaks in endemic countries. However, many laboratories lack proper tools for molecular diagnosis of DENV infection [52]. Although the vast majority of the patients included in the present study had been infected with DENV in Southeast Asia, some of them were Swedish residents returning from travel in Africa, the Caribbean, and South America, and they were all diagnosed with the virus by use of the DENV RT-PCR assay. This observation strongly indicates that the primers and probe used in our assay target genomes of DENV circulating in different regions. However, the assay has yet to be evaluated in clinical settings in endemic regions where other clinical profiles such as secondary infections may be overrepresented compared to our setting where we almost exclusively diagnose primary infections. Although we did not observe PCR inhibition in our clinical material, it can be valuable to include an inhibitory control [53] if less preserved samples are to be analyzed using the DENV RT-PCR.

Finally, the DENV RT-PCR assay showed 100% positive agreement with laboratory diagnosis performed using NS1 antigen detection tests. Viral RNA could be detected in samples obtained up to 5 days after disease onset and in more than half of the samples collected 6 to 9 days after appearance of symptoms. Hence the DENV RT-PCR assay is the method of choice during the first 5 days of disease, but after that period it should be supplemented with serology-based techniques.

## Supporting Information

S1 Figure **DENV genome sequence diversity.** Variability plot showing the percentage of sequences that deviate from the majority consensus at each nucleotide position. The gray line shows per position deviations and the black line shows ±10 positions unweighed moving average.
(TIF)

S2 Figure **Geographic and temporal distribution of DENV 3′ UTR sequences and their deviations at the DENV RT-PCR assay target site.** A total of 3021 DENV sequences covering the entire DENV RT-PCR binding site were found in the NCBI nucleotide collection (17 September 2014) of which 1125 were DENV-1 (A), 958 DENV-2 (B), 797 DENV-3 (C), and 141 DENV-4 (D). Perfect match (grey) corresponds to no mismatches in the primers or probe. Mild mismatches (green) corresponds to one or a few mismatches near the 5′ end of primers and perfect match to the probe. Moderate mismatches (yellow) corresponds to one or a few mismatches in nucleotides not positioned in the 3′ region of the primer and maximum one mismatch in the probe. Severe mismatch (red) corresponds to one mismatch close to the 3′ end in primers, several evenly distributed mismatches in the primer, or two mismatches in the probe. Fatal mismatch (blue) corresponds to mismatch in the final nucleotide in the 3′ end of primers or three or more mismatches in the probe. A) Of 1125 DENV-1 sequences covering the complete amplicon, seven have one mismatch in the probe. Two sequences (GenBank acc. no. FJ639735 and FJ639819) have two mismatches in the probe, the authors of these sequences report that the indels in the 3′ UTR have not been validated. B) Of 958 DENV-2 sequences covering the complete amplicon, eight have one mismatch in the probe, none of the sequences have more than one mismatch. C) Of the 797 DENV-3 sequences covering the complete amplicon, one has one mismatch in the probe. One sequence (GenBank acc. no. JQ922554), collection date 1963, has two mismatches in the probe. D) Of the 141 DENV-4 sequences covering the complete amplicon, one has one mismatch in the probe. The remaining DENV1-4 sequences covering the complete amplicon, presented in A–D, have perfect match with the probe. The sequences included in this analysis covers the complete amplicon and includes complete genomes, incomplete genomes, 3′ UTR sequences, and polyprotein sequences. For the design of primers and probe only complete genome sequences were used.
(PDF)

S3 Figure **Schematic illustration of the secondary structure of the DENV RT-PCR binding region in the 3′ UTR.** The structure is adapted from Friebe and Harris 2010 [43]. The binding sites of the primers and probe are highlighted.
(TIF)

S1 Table **Performance of the DENV RT-PCR assay on external control panels.**
(DOCX)

S2 Table **Serological and RT-PCR results days 1–9 after symptom onset.**
(DOCX)

S1 Checklist **STARD checklist.**
(DOC)

S1 Flow Diagram **STARD flow diagram.**
(PDF)

S1 Text **Virus propagation.**
(DOCX)

S2 Text **In vitro transcribed RNA sequences.**
(DOCX)

S1 File **Alignment of the 3′ UTR sequences of pathogenic flaviviruses.** The alignment was created using Clustal Omega v 1.2.0 [36]. The following 3′ UTR sequences are included: DENV1 (GenBank acc. no. NC_001477), DENV2 (GenBank acc. no. NC_001474), DENV3 (GenBank acc. no. NC_001475), DENV4 (GenBank acc. no. NC_002640), JEV (GenBank acc. no. NC_001437), St. Louis encephalitis virus (GenBank acc. no. NC_007580), TBEV (GenBank acc. no. NC_001672), USUV (GenBank acc. no. NC_006551), WNV (GenBank acc. no. NC_001563), YFV (GenBank acc. no. NC_002031), and ZIKV (GenBank acc. no. NC_012532).
(FA) Click here for additional data file.
