# Supplementary material for: Universal Single-Probe RT-PCR Assay for Diagnosis of Dengue Virus Infections
Source: PLoS Negl Trop Dis. 2014 Dec 18;8(12):e3416. doi: 10.1371/journal.pntd.0003416 (PMC4270494; doi:10.1371/journal.pntd.0003416)

Fig. S2A. DENV1

- Perfect match
- Mild mismatch
- Moderate mismatch
- Severe mismatch
- Fatal mismatch

Geographic distribution

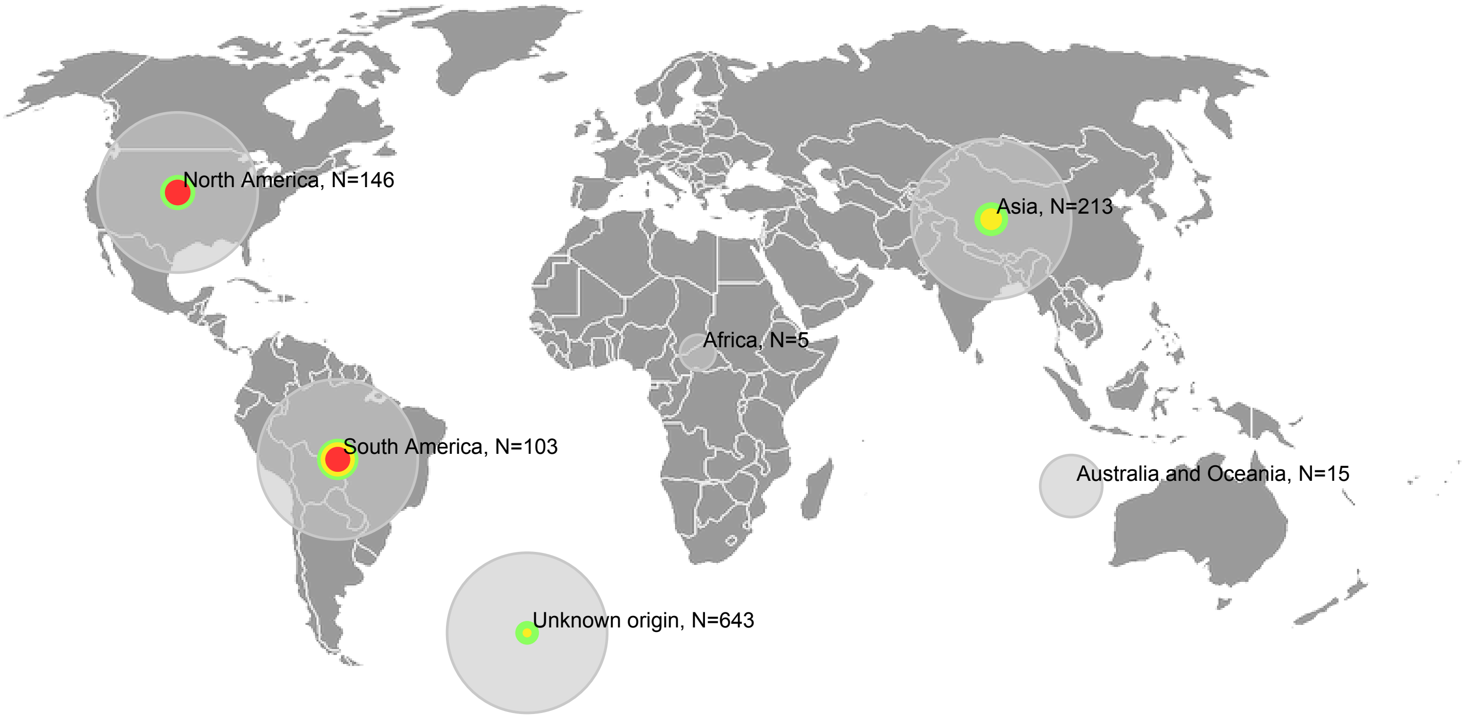

All sequences

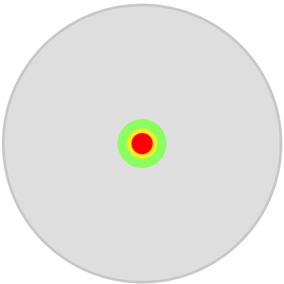

Perfect match: 97.2 %
Mild mismatch: 1.8 %
Moderate mismatch: 0.5 %
Severe mismatch: 0.4 %
Fatal mismatch: 0.0 %

Collection dates and mismatches

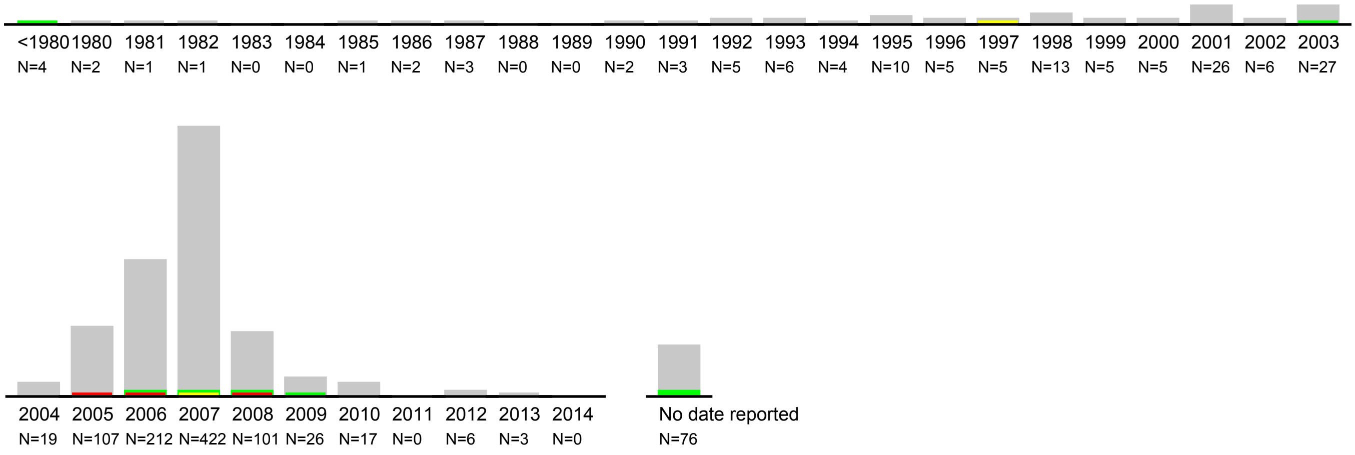

Fig. S2B. DENV2

Geographic distribution

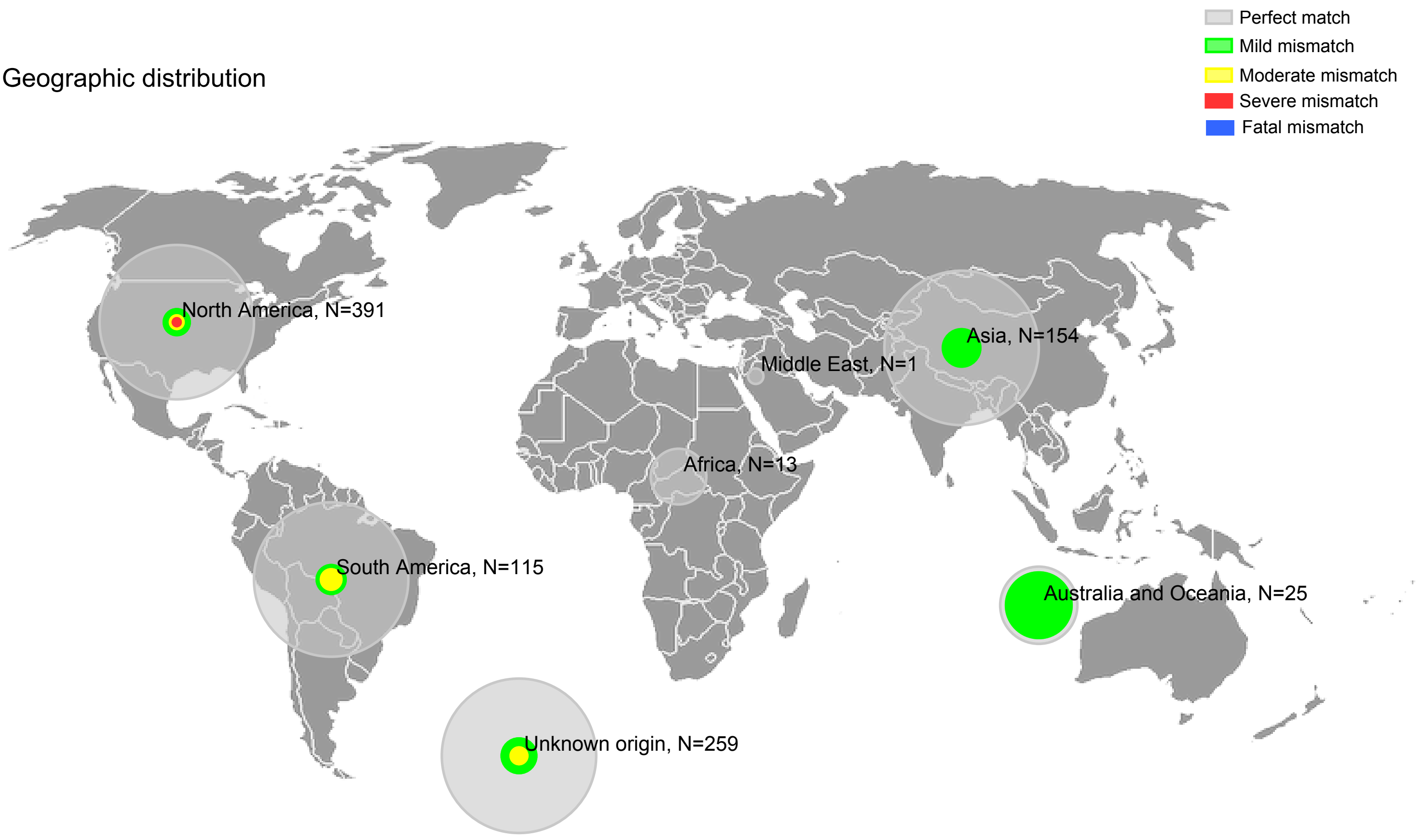

All sequences

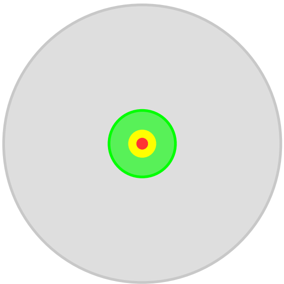

Perfect match: 94,3 %
Mild mismatch: 4,9 %
Moderate mismatch: 0,7 %
Severe mismatch: 0,1 %
Fatal mismatch: 0,0 %

Collection dates and mismatches

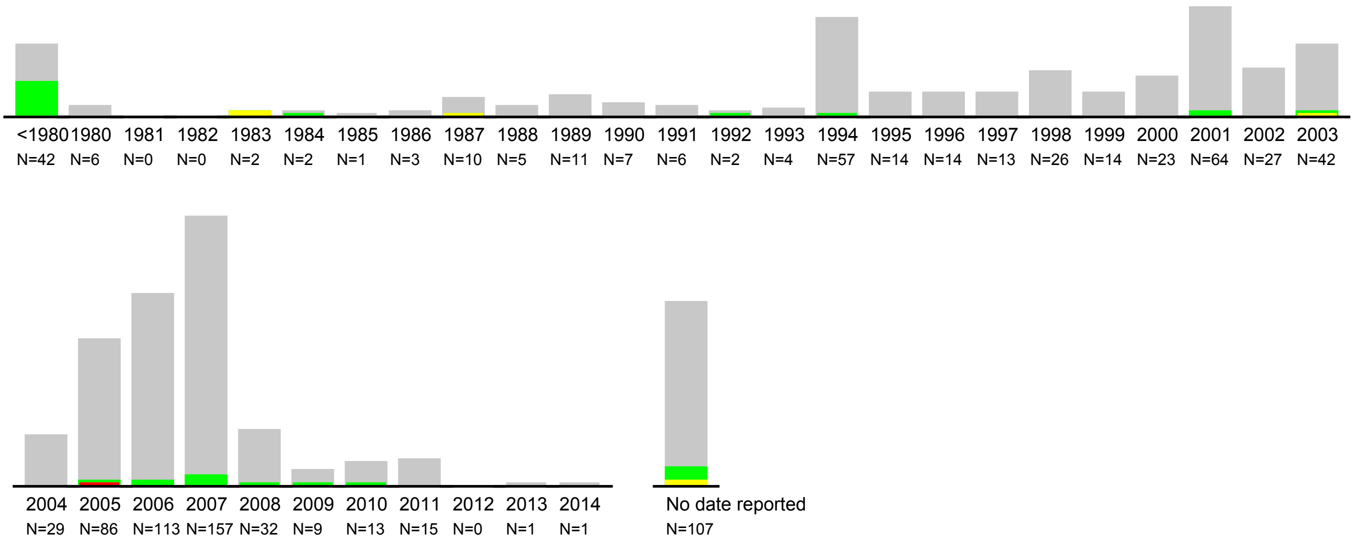

Fig. S2C. DENV3

Geographic distribution

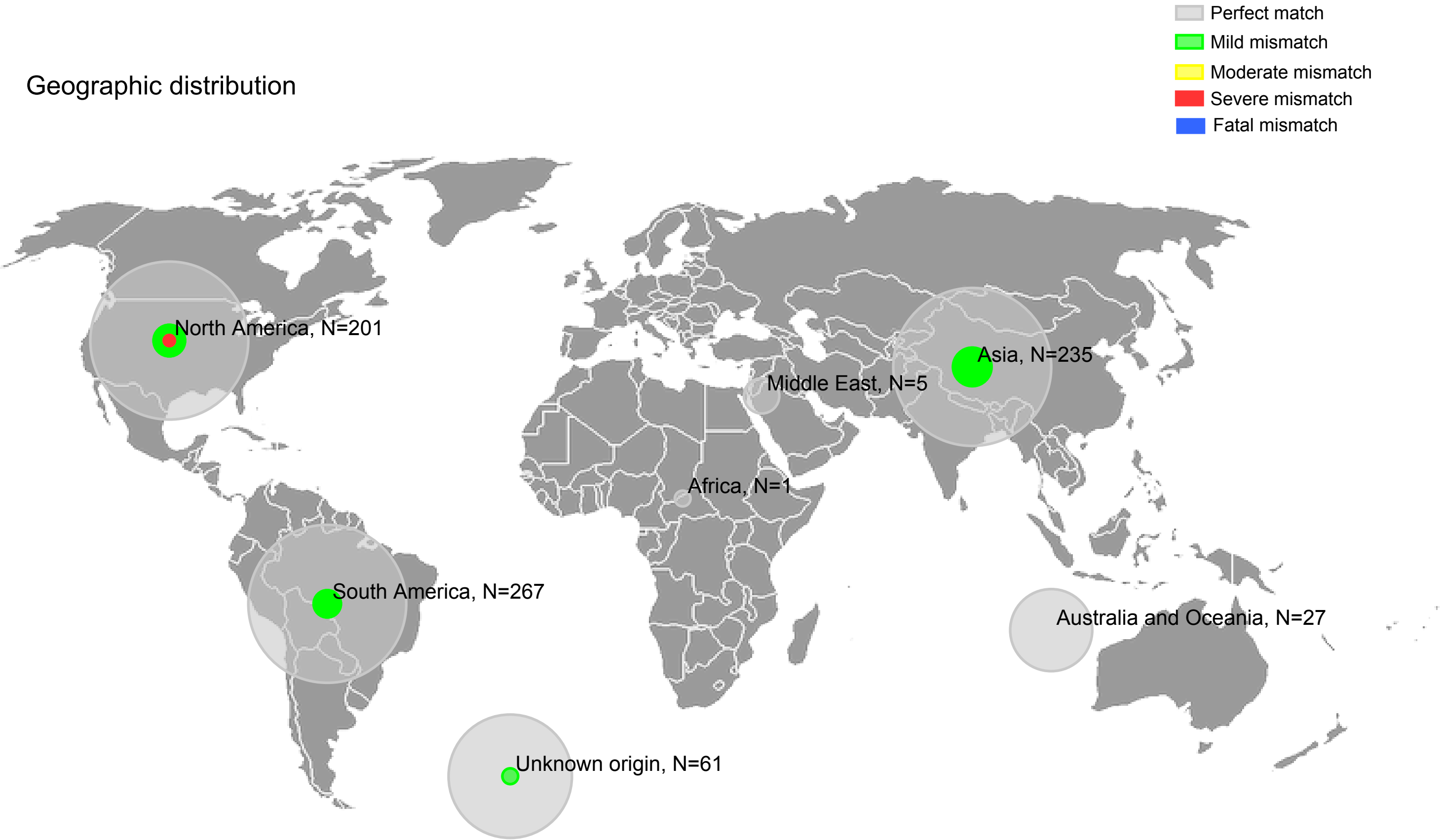

All sequences

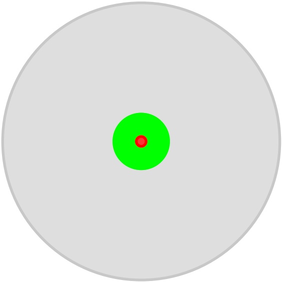

Perfect match: 96.1 %
Mild mismatch: 3.8 %
Moderate mismatch: 0.0 %
Severe mismatch: 0.1 %
Fatal mismatch: 0.0 %

Collection dates and mismatches

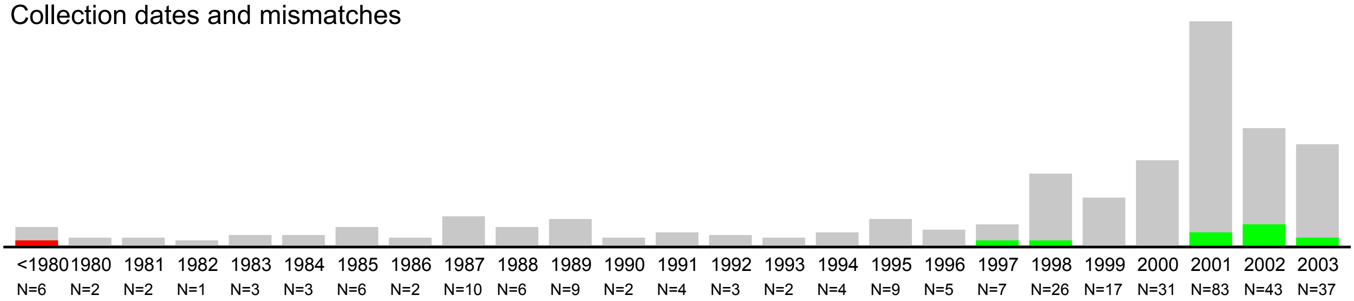

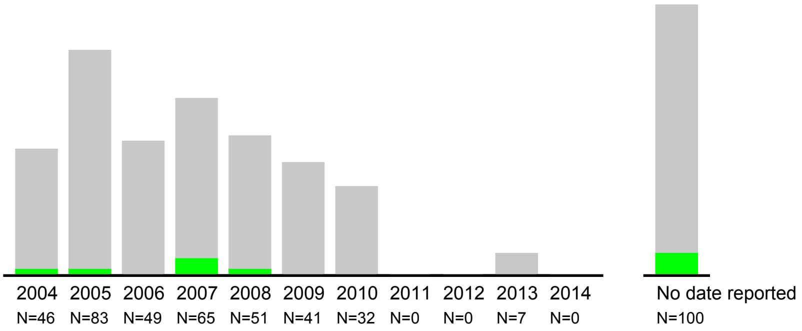

Fig. S2D. DENV4

Perfect match
Mild mismatch
Moderate mismatch
Severe mismatch
Fatal mismatch

Geographic distribution

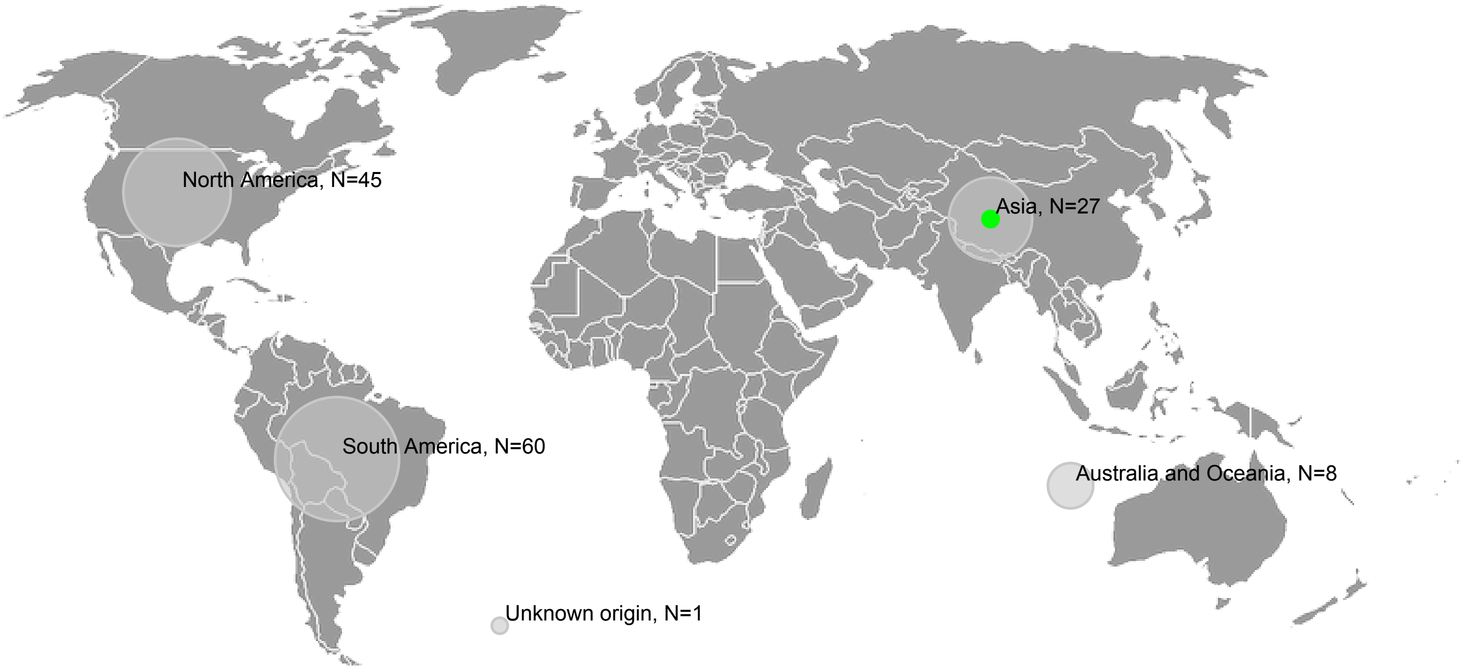

All sequences

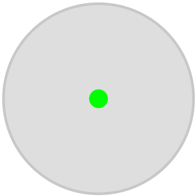

Perfect match: 99.3 %
Mild mismatch: 0.7 %
Moderate mismatch: 0.0 %
Severe mismatch: 0.0 %
Fatal mismatch: 0.0 %

Collection dates and mismatches

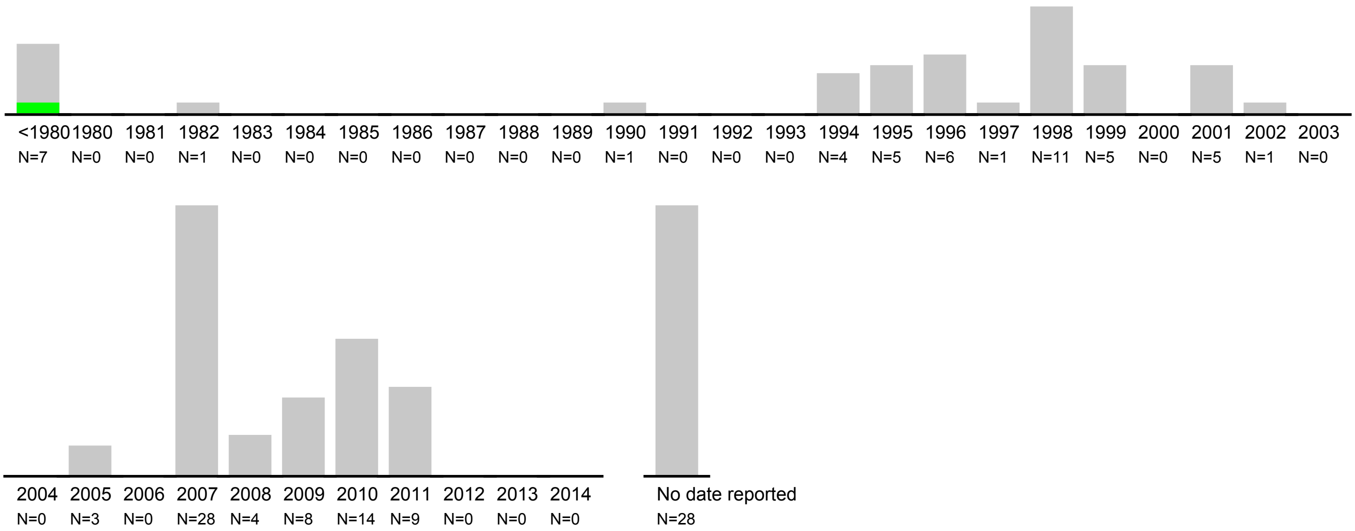

Supplement: S2 Figure — Geographic and temporal distribution of DENV 3′ UTR sequences and their deviations at the DENV RT-PCR assay target site. A total of 3021 DENV sequences covering the entire DENV RT-PCR binding site were found in the NCBI nucleotide collection (17 September 2014) of which 1125 were DENV-1 (A), 958 DENV-2 (B), 797 DENV-3 (C), and 141 DENV-4 (D). Perfect match (grey) corresponds to no mismatches in the primers or probe. Mild mismatches (green) corresponds to one or a few mismatches near the 5′ end of primers and perfect match to the probe. Moderate mismatches (yellow) corresponds to one or a few mismatches in nucleotides not positioned in the 3′ region of the primer and maximum one mismatch in the probe. Severe mismatch (red) corresponds to one mismatch close to the 3′ end in primers, several evenly distributed mismatches in the primer, or two mismatches in the probe. Fatal mismatch (blue) corresponds to mismatch in the final nucleotide in the 3′ end of primers or three or more mismatches in the probe. A) Of 1125 DENV-1 sequences covering the complete amplicon, seven have one mismatch in the probe. Two sequences (GenBank acc. no. FJ639735 and FJ639819) have two mismatches in the probe, the authors of these sequences report that the indels in the 3′ UTR have not been validated. B) Of 958 DENV-2 sequences covering the complete amplicon, eight have one mismatch in the probe, none of the sequences have more than one mismatch. C) Of the 797 DENV-3 sequences covering the complete amplicon, one has one mismatch in the probe. One sequence (GenBank acc. no. JQ922554), collection date 1963, has two mismatches in the probe. D) Of the 141 DENV-4 sequences covering the complete amplicon, one has one mismatch in the probe. The remaining DENV1-4 sequences covering the complete amplicon, presented in A–D, have perfect match with the probe. The sequences included in this analysis covers the complete amplicon and includes complete genomes, incomplete genomes, 3′ UTR sequences, and polypr [file pntd.0003416.s002.pdf]
