# Supplementary figures and images for: Universal Single-Probe RT-PCR Assay for Diagnosis of Dengue Virus Infections
Source: PLoS Negl Trop Dis. 2014 Dec 18;8(12):e3416. doi: 10.1371/journal.pntd.0003416 (PMC4270494; doi:10.1371/journal.pntd.0003416)

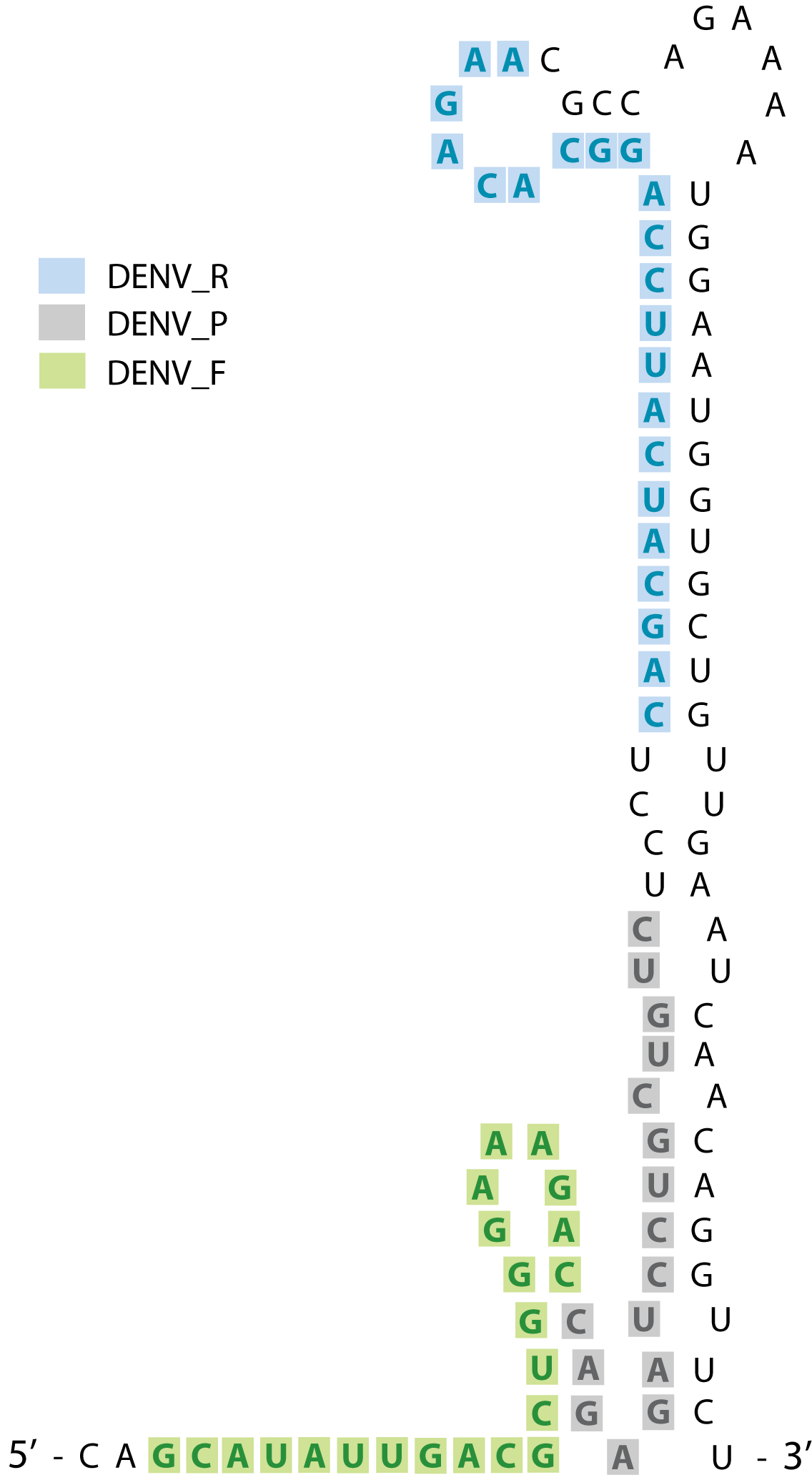

Supplement: S3 Figure — Schematic illustration of the secondary structure of the DENV RT-PCR binding region in the 3′ UTR. The structure is adapted from Friebe and Harris 2010 [43]. The binding sites of the primers and probe are highlighted. (TIF) [file pntd.0003416.s003.tif]

General example

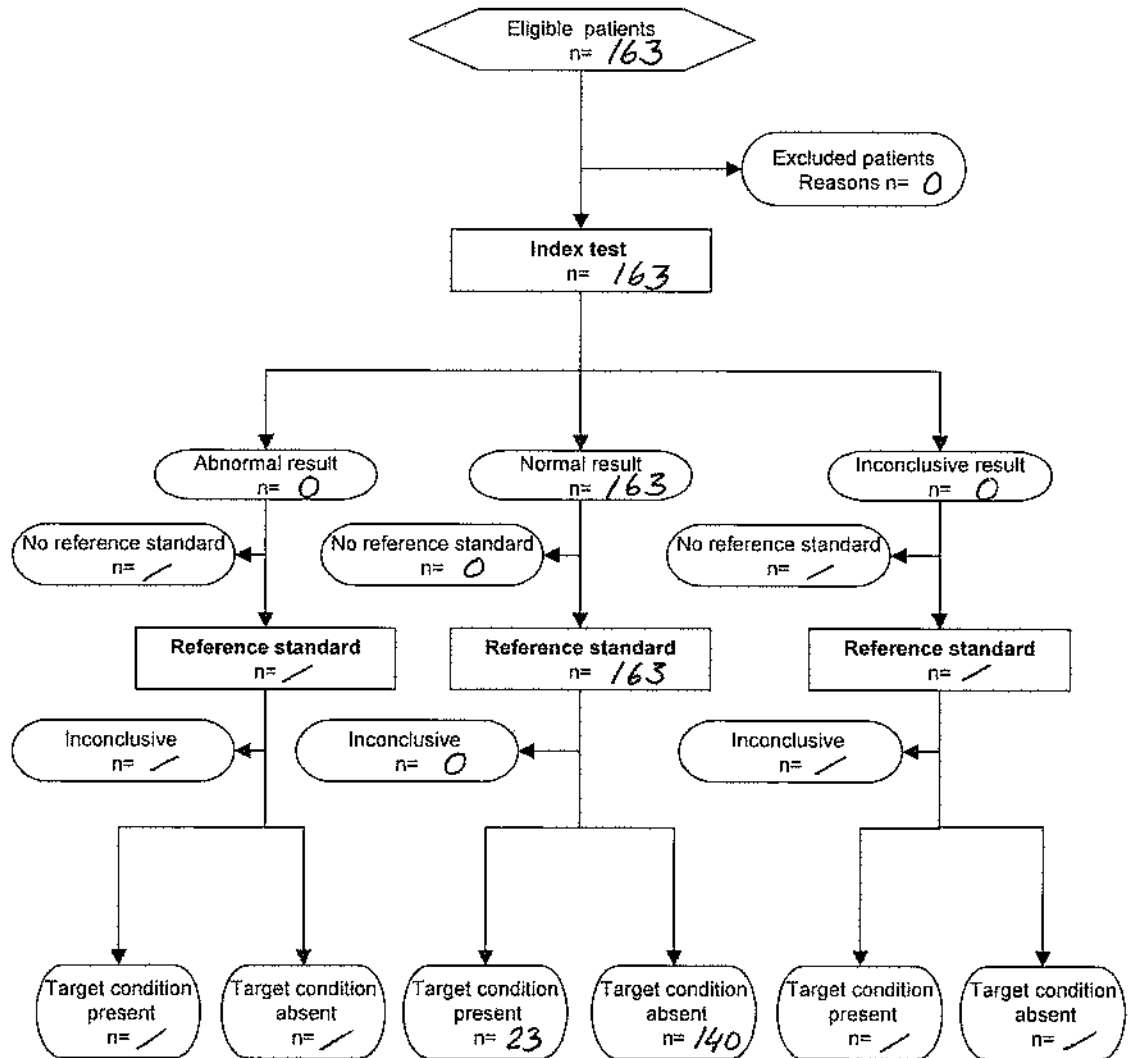

Supplement: S1 Flow Diagram — STARD flow diagram. (PDF) [file pntd.0003416.s007.pdf]
