# Supplementary material for: Universal Single-Probe RT-PCR Assay for Diagnosis of Dengue Virus Infections
Source: PLoS Negl Trop Dis. 2014 Dec 18;8(12):e3416. doi: 10.1371/journal.pntd.0003416 (PMC4270494; doi:10.1371/journal.pntd.0003416)
Supplement: S1 Table — Performance of the DENV RT-PCR assay on external control panels. (DOCX) [file pntd.0003416.s004.docx]

**Table S1.** Performance of the DENV RT-PCR assay on external control panels

| **Serotype** | **GCE/mL^1^** | **Mean C_q_–value of two replicates (standard deviation)** | | |
| --- | --- | --- | --- | --- |
|  |  | ***Panel 2011*** | ***Panel 2012*** | ***Panel 2013*** |
| DENV-1 | 10^6^ | 23.66 (0.06) | 23.46 (0.05) | 21.90 (0.00) |
| DENV-1 | 10^5^ | 26.61 (0.02) | 27.38 (0.09) | 25.61 (0.06) |
| DENV-1 | 10^5^ | 26.75 (0.04) | 26.90 (0.01) | 25.57 (0.03) |
| DENV-1 | 10^4^ | 29.59 (0.01) | 30.12 (0.06) | 32.77 (0.25) |
| DENV-1 | 10^3^ | 32.44 (0.01) | 32.68 (0.73) | 31.59 (0.13) |
| DENV-2 | 10^5^ | 30.90 (0.10) | 30.21 (0.06) | 30.74 (0.00) |
| DENV-2 | 10^4^ | 34.05 (0.21) | 32.79 (0.24) | 29.07 (0.21) |
| DENV-3 | 10^5^ | 30.03 (0.01) | 29.83 (0.09) | 28.36 (0.02) |
| DENV-3 | 10^4^ | 32.78 (0.28) | 32.18 (0.05) | 31.75 (0.20) |
| DENV-4 | 10^5^ | 29.45 (0.03) | 28.92 (0.02) | 27.13 (0.19) |
| Non-DENV flavivirus | na | nd | nd | nd |
| Negative control | na | nd | nd | nd |

^1^Genome copy equivalents (GCE)/mL reported by QCMD.

na = not available; nd = not detected
