# Supplementary material for: Universal Single-Probe RT-PCR Assay for Diagnosis of Dengue Virus Infections
Source: PLoS Negl Trop Dis. 2014 Dec 18;8(12):e3416. doi: 10.1371/journal.pntd.0003416 (PMC4270494; doi:10.1371/journal.pntd.0003416)
Supplement: S2 Table — Serological and RT-PCR results days 1–9 after symptom onset. (DOCX) [file pntd.0003416.s005.docx]

| **Table S2. Serological and RT-PCR results days 1-9 after symptom onset** | | | | | |
| --- | --- | --- | --- | --- | --- |
| **Sample** | **GCE/mL^1^** | **NS1 ag.** | **IgM** | **IgG** | **Serotype^2^** |
| *Samples collected ≤ 3 days after symptom onset* | | | | | |
| 1 | 5.1 x 10^5^ | P | N | <40 | DENV-1 |
| 2 | 9.1 x 10^6^ | P | 46 | <40 | DENV-1 |
| 3 | 1.7 x 10^8^ | P | 20 | 320 | DENV-2 |
| 4 | 4.8 x 10^6^ | P | N | <40 | DENV-3 |
| 5 | 3.2 x 10^5^ | P | 38 | <40 | DENV-2 |
| *Samples collected 4 days after symptom onset* | | | | | |
| 6 | 9.2 x 10^5^ | P | 135 | 160 | DENV-4 |
| 7 | 2.7 x 10^7^ | P | 26 | 160 | DENV-1 |
| 8 | 3.7 x 10^6^ | P | 20 | 40 | DENV-1 |
| 9 | 4.4 x 10^4^ | P | 62 | 320 | DENV-1 |
| 10 | 2.2 x 10^5^ | P | 22 | 640 | DENV-1 |
| 11 | 1.0 x 10^7^ | P | 64 | <40 | DENV-1 |
| 12 | 1.8 x 10^6^ | P | 20 | <40 | DENV-3 |
| 13 | 2.9 x 10^4^ | P | 102 | >640 | DENV-1 |
| 14 | 1.5 x 10^5^ | P | 70 | <40 | DENV-1 |
| 15 | 2.1 x 10^4^ | P | 144 | <40 | DENV-3 |
| *Samples collected 5 days after symptom onset* | | | | | |
| 16 | 2.4 x 10^4^ | P | N | <40 | DENV-3 |
| 17 | 4.0 x 10^4^ | P | 129 | >640 | DENV-1 |
| 18 | 1.9 x 10^5^ | P | 63 | >640 | DENV-4 |
| 19 | 2.3 x 10^3^ | P | 49 | >640 | NA^3^ |
| 20 | 3.9 x 10^4^ | P | 90 | 1280 | DENV-1 |
| 21 | 4.7 x 10^3^ | P | 135 | 640 | DENV-2 |
| 22 | 5.9 x 10^4^ | P | 46 | <40 | DENV-1 |
| *Samples collected 6 days after symptom onset* | | | | | |
| 23 | 1.0 x 10^3^ | P | 75 | 640 | DENV-2 |
| 24 | 2.9 x 10^3^ | P | 150 | <40 | DENV-1 |
| 25 | 2.7 x 10^3^ | P | 129 | <40 | DENV-1 |
| 26 | 2.6 x 10^4^ | P | 71 | >640 | DENV-1 |
| 27 | 1.3 x 10^4^ | P | 152 | 640 | DENV-2 |
| 28 | nd | N | 20 | 160 | nd |
| 29 | 7.7 x 10^3^ | P | 97 | 40 | NA^3^ |
| 30 | 2.6 x 10^3^ | P | 113 | >640 | DENV-1 |
| 31 | nd | N | 72 | >640 | nd |
| 32 | 5.8 x 10^2^ | P | 95 | <40 | DENV-1 |
| 33 | 7.7 x 10^3^ | P | 148 | <40 | DENV-1 |
| *Samples collected 7 days after symptom onset* | | | | | |
| 34 | nd | N | 82 | 640 | nd |
| 35 | 1.1 x 10^3^ | P | 166 | 640 | DENV-1 |
| 36 | 1.9 x 10^3^ | P | 112 | 160 | DENV-1 |
| 37 | 4.1 x 10^2^ | P | 139 | >640 | DENV-2 |
| 38 | 3.3 x 10^3^ | N | 140 | >640 | DENV-1 |
| 39 | 1.1 x 10^3^ | P | 144 | 40 | nd |
| 40 | nd | N | 74 | 640 | nd |
| 41 | 5.4 x 10^3^ | P | 104 | >640 | DENV-1 |
| 42 | 7.5 x 10^2^ | P | 142 | <40 | nd |
| 43 | 8.9 x 10^3^ | P | 109 | <40 | DENV-3 |
| 44 | 1.0 x 10^4^ | P | 89 | <40 | DENV-3 |
| 45 | 6.8 x 10^2^ | P | 113 | 2560 | nd |
| *Samples collected 8 days after symptom onset* | | | | | |
| 46 | nd | N | 94 | 640 | nd |
| 47 | 8.6 x 10^2^ | P | 37 | 160 | DENV-1 |
| 48 | 2.1 x 10^2^ | P | 98 | >640 | nd |
| 49 | 4.7 x 10^3^ | P | 101 | >640 | DENV-1 |
| 50 | nd | N | 153 | >640 | nd |
| 51 | 7.5 x 10^5^ | P | 159 | 640 | DENV-1 |
| 52 | 6.9 x 10^3^ | P | 65 | 640 | nd |
| 53 | 3.4 x 10^3^ | P | 87 | <40 | nd |
| *Samples collected 9 days after symptom onset* | | | | | |
| 54 | nd | N | 45 | >640 | nd |
| 55 | 8.0 x 10^3^ | P | 63 | 40 | DENV-1 |
| 56 | nd | N | 143 | >640 | nd |
| 57 | 3.7 x 10^2^ | P | 126 | >640 | DENV-1 |
| 58 | 2.0 x 10^2^ | N | 104 | >640 | nd |
| 59 | 4.4 x 10^2^ | P | 91 | 640 | nd |
| 60 | nd | N | 142 | 160 | nd |

^1^Genome copy equivalents /mL determined by the DENV RT-PCR Assay.

^2^The DENV serotype was determined using the CDC DENV-1-4 Real Time RT-PCR Assay in a singleplex format.

^3^No additional material was available for DENV serotyping.

nd = not detected
