# Supplementary material for: Universal Single-Probe RT-PCR Assay for Diagnosis of Dengue Virus Infections
Source: PLoS Negl Trop Dis. 2014 Dec 18;8(12):e3416. doi: 10.1371/journal.pntd.0003416 (PMC4270494; doi:10.1371/journal.pntd.0003416)
Supplement: S1 Text — Virus propagation. (DOCX) [file pntd.0003416.s008.docx]

**Text S1: Virus propagation**

Virus, except for ZIKV and Hantaviruses, was propagated in Vero cells (ATCC: CCL-81) in medium 199 supplemented with 5% heat-inactivated fetal bovine serum (FBS), 1 mM HEPES, and 1% PEST. DOBV, SEOV, and HTNV were propagated in Vero E6 cells (ATCC: CRL-1586) in DMEM supplemented with 5% FBS and 1% PEST. Cells were maintained at 37°C in a humidified atmosphere containing 5% CO_2_. ZIKV was propagated in *Aedes albopictus* C6/36 cells (ATCC: CRL-1660) in Leibovitz’s L-15 medium supplemented with 5% FBS, 2% tryptose phosphate, and 1% PEST at 28°C in a humidified atmosphere containing 0% CO_2_. All reagents were purchased from Life Technologies.
