# Supplementary material for: Universal Single-Probe RT-PCR Assay for Diagnosis of Dengue Virus Infections
Source: PLoS Negl Trop Dis. 2014 Dec 18;8(12):e3416. doi: 10.1371/journal.pntd.0003416 (PMC4270494; doi:10.1371/journal.pntd.0003416)
Supplement: S2 Text — In vitro transcribed RNA sequences. (DOCX) [file pntd.0003416.s009.docx]

**Text S2: In vitro transcribed RNA sequences**

RNA[DENV_1-3]

AACAATAAACAGCATATTGACGCTGGGAGAGACCAGAGATCCTGCTGTCTCTACAGCATCATTCCAGGCACAGAACGCCAGAAAATGG

RNA[DENV_4]

CACAAAAACAGCATATTGACGCTGGGAAAGACCAGAGATCCTGCTGTCTCTACAACATCAATCCAGGCACAGAGCGCCGCAAGA
